# Effect of intracranial pressure monitoring-based therapy on clinical efficacy and serum levels in neurosurgical patients with severe intracerebral hemorrhage

**DOI:** 10.3389/fneur.2026.1943765

**Published:** 2026-09-11

**Authors:** Lizhi Jiang, Jiawei Yu, Bing Chen, Jia Wang, Yu Zhang

**Affiliations:** 1 Taihe Hospital, Hubei University of Medicine, Shiyan, Hubei, China; 2 Yunyang County People's Hospital, Chongqing, China; 3 Yunxi County People's Hospital, Shiyan, Hubei, China

**Keywords:** external ventricular drain, Glasgow Outcome Scale, intracranial hematoma evacuation, intracranial pressure monitoring, neurosurgery, propensity score matching, severe intracerebral hemorrhage

## Abstract

**Objective:**

To investigate the effect of intracranial pressure (ICP) monitoring combined with hematoma evacuation on clinical efficacy and serum markers in neurosurgical patients with severe intracerebral hemorrhage.

**Methods:**

A total of 120 patients with severe intracerebral hemorrhage treated in the Department of Neurology, Taihe Hospital, Hubei University of Medicine from January 2025 to January 2026 were retrospectively enrolled. According to whether postoperative ICP monitoring was performed, the patients were divided into an ICP monitoring group (hematoma evacuation + external ventricular drain-based ICP monitoring) and a control group (hematoma evacuation alone). Propensity score matching at a 1:1 ratio was used to balance baseline characteristics, and 57 patients were ultimately included in each group. The surgical indicators, 3-month favorable outcome defined as Glasgow Outcome Scale score 4–5, neurological deficit scores, serum levels of endothelin-1 (ET-1), matrix metalloproteinase-9 (MMP-9), advanced oxidation protein products (AOPP), cortisol (Cor), and norepinephrine (NE), activity of daily living (ADL) score, and complication rates were compared between the two groups.

**Results:**

After matching, there were no significant differences in intraoperative blood loss and evacuated hematoma volume between the two groups (both *p* > 0.05). The operation time of the ICP monitoring group was longer than that of the control group (*p* < 0.05), while the hematoma evacuation time and hospital stay were significantly shorter (both *p* < 0.05). The 3-month favorable outcome rate in the ICP monitoring group was 87.72% (50/57), which was significantly higher than 49.12% (28/57) in the control group (*p* < 0.05). After treatment, the Chinese Stroke Scale (CSS) scores and National Institutes of Health Stroke Scale (NIHSS) scores in the ICP monitoring group were both lower than those in the control group (both *p* < 0.05); the serum levels of ET-1, MMP-9, AOPP, Cor, and NE in the ICP monitoring group were also significantly lower than those in the control group (all *p* < 0.05). The ADL score of the ICP monitoring group was significantly higher than that of the control group (*p* < 0.05). The overall complication rate was 7.02% (4/57) in the ICP monitoring group, which was significantly lower than 22.81% (13/57) in the control group (*p* < 0.05).

**Conclusion:**

Intracranial pressure monitoring combined with hematoma evacuation was associated with better clinical outcome, lower serum levels of ET-1, MMP-9, AOPP, and stress hormones, fewer complications, and greater independence in daily living. However, the observational design limits causal inference, and the external ventricular drain may have contributed both monitoring and therapeutic drainage effects that cannot be completely separated in this study.

## 1 Introduction

Spontaneous intracerebral hemorrhage is one of the stroke subtypes with the highest rates of mortality and disability. In severe cases, hematoma compression and secondary cerebral edema often lead to a rapid increase in intracranial pressure, which in turn causes decreased cerebral perfusion, brain tissue shift, and even cerebral herniation, threatening life (1, 2). Although emergency craniotomy for hematoma evacuation can rapidly relieve mechanical compression, patients still face multiple postoperative challenges, including the continuous progression of perihematomal edema, intensified oxidative stress, activated inflammatory cascades, and recurrent intracranial hypertension, which significantly impede neurological recovery and affect long-term prognosis (3–5).

Traditional postoperative management largely relies on clinical experience to adjust ICP-lowering strategies, supplemented by intermittent cranial imaging assessments, making it difficult to capture dynamic fluctuations in ICP in real time and easily resulting in delayed intervention or overtreatment. In contrast, continuous ICP monitoring can provide precise and continuous physiological parameters, offering an objective basis for stepwise ICP management. This strategy has been widely adopted in severe traumatic brain injury (6, 7). However, its application value following surgery for severe intracerebral hemorrhage, particularly the specific mechanisms underlying neurological improvement, still lacks systematic evidence.

Notably, elevated ICP is not merely a physical pathological manifestation but also triggers a series of molecular cascades: vasoactive substances such as endothelin-1 and matrix metalloproteinase-9 are activated, large amounts of inflammatory mediators are released, and the sympathetic-adrenomedullary system is intensely stimulated, leading to a significant increase in stress hormones such as cortisol and norepinephrine, which further aggravates neuronal injury. It remains unclear whether precise intervention guided by ICP monitoring can effectively regulate the expression of these serum factors, thereby achieving neuroprotection and prognosis optimization.

The present retrospective study was designed to evaluate the association between combined ICP monitoring and hematoma evacuation and clinical outcomes and serum markers in patients with severe intracerebral hemorrhage, rather than to establish causal mechanisms.

## 2 Materials and methods

### 2.1 Study participants

This study retrospectively enrolled 120 patients with severe intracerebral hemorrhage who were hospitalized in the Department of Neurology, Taihe Hospital, Hubei University of Medicine between January 2025 and January 2026. All cases were confirmed by cranial CT and met the following inclusion criteria: hematoma volume not less than 30 mL, Glasgow Coma Scale score between 5 and 12, age ranging from 18 to 75 years, first-ever onset, and receipt of emergency craniotomy for hematoma evacuation. Exclusion criteria included: hemorrhage caused by trauma, aneurysm rupture, or vascular malformation; progression to advanced cerebral herniation with bilateral fixed and dilated pupils; concomitant severe cardiac, hepatic, or renal failure; presence of uncorrectable coagulopathy or ongoing anticoagulant therapy; and patients with severe disability resulting from prior stroke.

Patients who underwent ICP monitoring were selected at the discretion of the treating neurosurgeon according to clinical indication, including suspected or anticipated intracranial hypertension; this potential indication bias was addressed by propensity score matching.

The entire study was conducted in accordance with the ethical principles of the Declaration of Helsinki and was approved by the Ethics Committee of Taihe Hospital, Hubei University of Medicine (Approval No.: TH20240812). Given the retrospective nature of the study, informed consent was waived, and patient privacy was strictly protected.

### 2.2 Treatment methods

Patients in the control group received conventional minimally invasive trephination craniopuncture for hematoma evacuation. Under general anesthesia, the surgeon determined the puncture trajectory based on preoperative CT localization, used a trephination device to percutaneously puncture into the hematoma cavity, inserted a drainage tube, aspirated the liquid hematoma under negative pressure, and repeatedly irrigated with sterile normal saline until the drainage fluid became clear. To promote the dissolution of residual blood clots, 5,000 IU of urokinase was subsequently injected into the hematoma cavity, the drainage tube was clamped for 4 h, and then continuous drainage was reopened. Cranial CT was reexamined within 24 to 48 h postoperatively, and the drainage tube was removed once the residual hematoma volume was less than 5 mL or largely absorbed.

In the ICP monitoring group, on the basis of completing the same hematoma evacuation procedure as described above, an external ventricular drain (EVD) was inserted into the frontal horn of the lateral ventricle and used both for continuous ICP recording and for therapeutic cerebrospinal fluid drainage.

During the procedure, a scalp incision was made within the hairline on the ipsilateral side, approximately 2.5 cm lateral to the midline. After drilling a burr hole with a cranial drill, a ventricular puncture needle was slowly advanced in the direction pointing toward the midpoint of the imaginary line connecting the bilateral external auditory canals. Once cerebrospinal fluid overflowed from the needle core, the catheter was gently advanced approximately 2 cm further, so that its tip was stably positioned within the frontal horn of the lateral ventricle. The catheter was externalized through a subcutaneous tunnel, fixed with silk sutures, and connected to a closed drainage and monitoring system. The pressure sensor was connected to a multi-channel monitor, and after zero calibration at the level of the external auditory canal, real-time recording of ICP changes was initiated.

Postoperative management differed between the two groups. In the control group, decisions regarding osmotic therapy, sedation, and re-imaging were made by the treating team based on clinical signs, neurologic examination, and serial CT findings, without real-time ICP data. In the ICP monitoring group, a stepwise ICP management algorithm was applied: (1) head elevation to 30° and optimization of sedation and analgesia; (2) cerebrospinal fluid drainage through the EVD if ICP remained >20 mmHg for more than 5 min; (3) hyperosmolar therapy with 20% mannitol (0.25–0.5 g/kg) or 3% hypertonic saline; and (4) immediate repeat CT if ICP remained refractory or neurologic deterioration occurred. The treatment goal was to maintain ICP ≤ 20 mmHg and cerebral perfusion pressure ≥70 mmHg, with blood pressure managed according to current guidelines for spontaneous intracerebral hemorrhage (3, 6).

### 2.3 Propensity score matching

To control for potential confounding bias introduced by non-randomized grouping, propensity score matching (PSM) was employed to balance the baseline characteristics between the two groups. With treatment group assignment as the dependent variable, age, sex, preoperative hematoma volume, Glasgow Coma Scale score, hemorrhage location (basal ganglia, thalamus, or lobar), and history of hypertension were included as covariates to construct a logistic regression model for estimating the matching probability of each patient. Subsequently, a 1:1 nearest-neighbor matching algorithm was applied with a caliper value set at 0.02 to ensure a high degree of consistency in covariate distribution between the matched groups. After matching, 57 patients were included in each of the ICP monitoring and control groups for the final analysis, achieving good balance in key clinical characteristics between the two groups.

### 2.4 Outcome measures

The study systematically recorded operation time, intraoperative blood loss, evacuated hematoma volume, hematoma evacuation time, and hospital stay to comprehensively evaluate the implementation efficiency of the intervention and the clinical burden on patients.

The 3-month neurological outcome was graded using the Glasgow Outcome Scale (GOS) (8): good recovery (GOS 5), moderate disability (GOS 4), severe disability (GOS 3), vegetative state (GOS 2), and death (GOS 1). The favorable outcome rate was defined as the proportion of patients with GOS 4–5. Because of the small number of deaths, mortality was included in the unfavorable outcome category but was not analyzed as a separate endpoint.

The degree of neurological deficit was assessed using both the Chinese Stroke Scale (CSS) and the National Institutes of Health Stroke Scale (NIHSS). The CSS covers dimensions including consciousness, facial palsy, and limb weakness, with a total score ranging from 0 to 45, corresponding to mild (0–15 points), moderate (16–30 points), and severe (31–45 points) deficits; the NIHSS provides a comprehensive assessment across 11 dimensions including consciousness, gaze, sensation, language, and motor function, with a total score of 0 to 42, where higher scores indicate more severe neurological injury.

For serological testing, fasting venous blood samples were collected before surgery (baseline) and on postoperative day 7 before routine morning medications. After centrifugation, serum was stored at −80 °C. Enzyme-linked immunosorbent assay (ELISA) was used to measure the concentrations of endothelin-1, matrix metalloproteinase-9, advanced oxidation protein products, cortisol, and norepinephrine, reflecting the activation status of inflammation, oxidative stress, and the stress axis.

Activity of daily living was evaluated using the modified Barthel Index (ADL), covering 10 basic activities including eating, mobility, toileting, dressing, bathing, walking, and balance, with a total score of 100, where higher scores indicate greater functional independence.

Postoperative complications were recorded separately for the external ventricular drain site and the hematoma puncture site when applicable. Scalp exudation was defined as persistent serous or serosanguineous leakage at the puncture/drain site requiring dressing changes beyond 72 h or additional suturing. Rebleeding was defined as new or expanded hemorrhage on CT with clinical deterioration. Intracranial infection was defined as culture-positive cerebrospinal fluid or clinical ventriculitis/meningitis according to accepted criteria. The overall incidence was used as the primary safety endpoint for statistical analysis.

### 2.5 Statistical methods

SPSS 26.0 software was used for data analysis. The normality of continuous variables was assessed using the Shapiro–Wilk test. Normally distributed data were expressed as mean ± standard deviation, and intergroup comparisons were performed using the independent samples *t*-test; non-normally distributed data were expressed as median and interquartile range, and the Mann–Whitney *U* test was applied. Categorical data were expressed as counts and percentages, and intergroup comparisons were conducted using the *χ*^2^ test or Fisher’s exact test. A *p*-value < 0.05 was considered statistically significant.

## 3 Results

### 3.1 Baseline characteristics

After propensity score matching, there were no statistically significant differences between the ICP monitoring group and the control group in terms of age, sex, hematoma volume, Glasgow Coma Scale score, hemorrhage location, and history of hypertension (all *p* > 0.05), indicating comparability between the two groups (see Table 1).

**Table 1:** Comparison of baseline characteristics between the two groups after matching.

Characteristic	ICP monitoring group	Control group	*t/x^2^*	*P*
Number of cases	57	57	–	–
Gender [example (%)]			0.15	0.702
Male	35(61.4)	33(57.9)		
Female	22(38.6)	24(42.1)		
Age (years)	56.2 ± 9.8	55.8 ± 10.1	0.21	0.834
Preoperative bleeding volume (mL)	45.7 ± 8.5	46.2 ± 9.0	0.30	0.765
Preoperative GCS score (points)	8.2 ± 1.6	8.0 ± 1.7	0.64	0.523
Bleeding site [example (%)]			0.31	0.858
Basal ganglia	36(63.2)	34(59.6)		
Thalamus	12(21.1)	13(22.8)		
Lobes	9 (15.8)	10(17.5)		
History of hypertension [case (%)]	41(71.9)	43(75.4)	0.18	0.672

GCS, Glasgow Coma Scale; no statistically significant differences were observed in any baseline characteristics between the two groups (all *p* > 0.05), confirming comparability.

### 3.2 Surgical indicators

There were no statistically significant differences in intraoperative blood loss and evacuated hematoma volume between the two groups (158.45 ± 11.22 vs. 163.87 ± 15.78 mL, 38.11 ± 3.25 vs. 35.90 ± 4.44 mL, all *p* > 0.05). The operation time of the ICP monitoring group was significantly longer than that of the control group (198.11 ± 24.98 vs. 144.58 ± 23.22 min), while the hematoma evacuation time and hospital stay were significantly shorter in the ICP monitoring group than in the control group (7.13 ± 0.28 vs. 11.22 ± 1.13 d, 11.18 ± 1.14 vs. 16.10 ± 1.32 d, all *p* < 0.05) (see Table 2).

**Table 2:** Comparison of surgical indicators between the two groups.

Indicator	ICP monitoring group	Control group	*t*	*P*
Number Of Cases	57	57	–	–
Surgical duration (min)	198.11 ± 24.98	144.58 ± 23.22	11.85	<0.001
Intraoperative bleeding volume (mL)	158.45 ± 11.22	163.87 ± 15.78	−1.92	0.057
Evacuated hematoma volume (mL)	38.11 ± 3.25	35.90 ± 4.44	2.91	0.055
Hematoma evacuation time (d)	7.13 ± 0.28	11.22 ± 1.13	−26.51	<0.001
Hospitalization time (d)	11.18 ± 1.14	16.10 ± 1.32	−20.36	<0.001

### 3.3 Clinical efficacy

**Figure 1:**
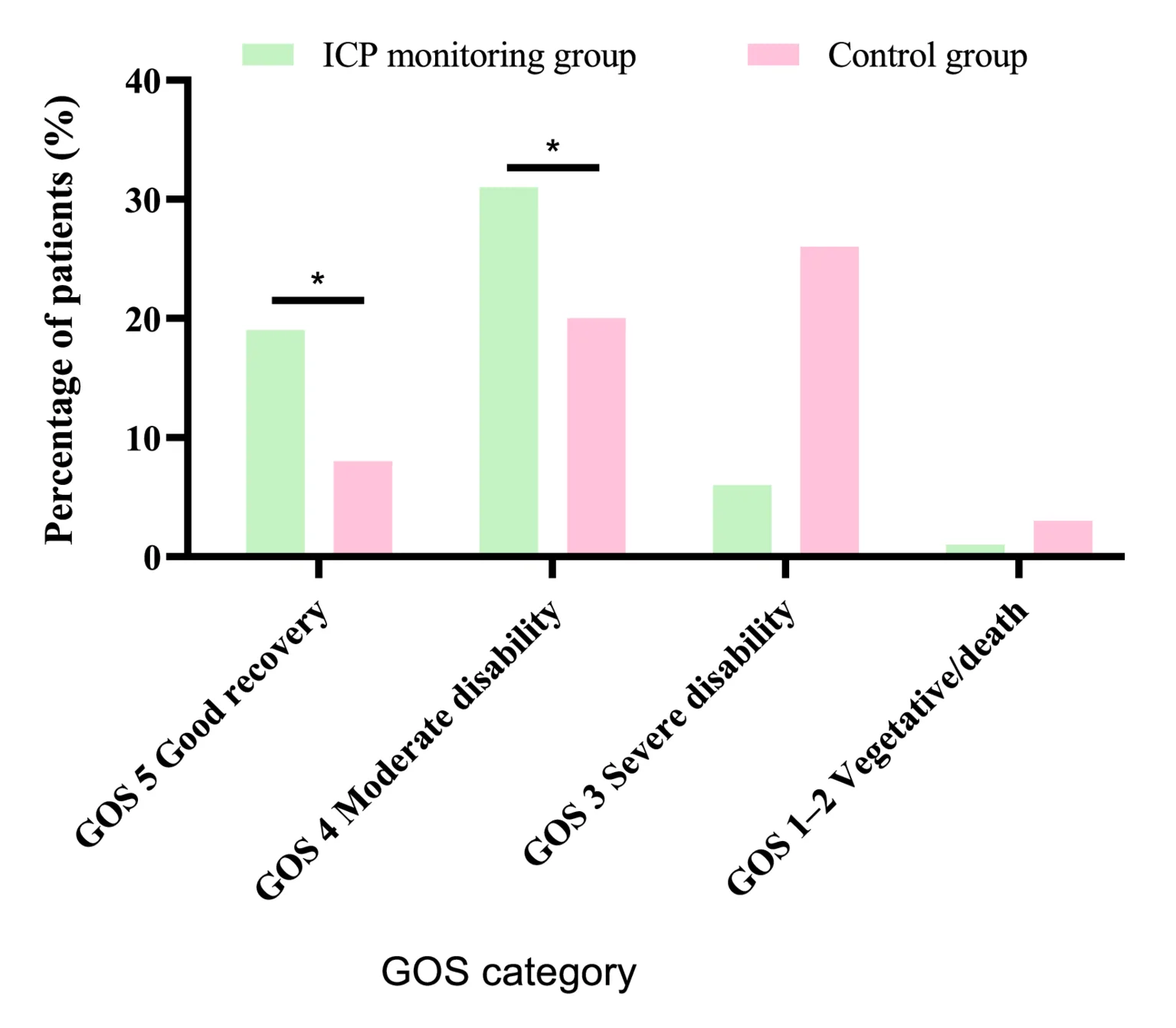
Three-month Glasgow Outcome Scale distribution in the ICP monitoring and control groups. Bars represent the percentage of patients within each Glasgow Outcome Scale (GOS) category. GOS 1 and GOS 2 were combined because of small numbers. Favorable outcome was defined as GOS 4–5. *p < 0.05 for the between-group comparison of favorable outcome.

The 3-month favorable outcome rate, defined as the proportion of patients with GOS 4–5, was 87.72% (50/57) in the ICP monitoring group and 49.12% (28/57) in the control group (*χ*^2^ = 21.86, *p* < 0.001). The remaining patients had GOS 1–3; mortality (GOS 1) was included in the unfavorable outcome category but was not reported separately because of the small number of events and the retrospective nature of the study (see Figure 1).

### 3.4 Neurological function scores

Before surgery, there were no statistically significant differences in CSS and NIHSS scores between the two groups (*p* > 0.05). After treatment, the CSS and NIHSS scores in the ICP monitoring group were both significantly lower than those in the control group (16.24 ± 1.77 vs. 24.01 ± 1.89, 18.22 ± 1.87 vs. 26.74 ± 2.13, all *p* < 0.05) (see Table 3).

**Table 3:** Comparison of neurological function scores between the two groups.

Scale	Time	ICP monitoring group	Control group	*t*	*P*
Number of cases	–	57	57	–	–
CSS (Score)	Before surgery	35.12 ± 3.45	34.89 ± 3.62	0.35	0.727
	After treatment	16.24 ± 1.77	24.01 ± 1.89	−21.88	<0.001
NIHSS (Score)	Before surgery	38.45 ± 3.11	38.12 ± 3.34	0.55	0.584
	After treatment	18.22 ± 1.87	26.74 ± 2.13	−21.14	<0.001

### 3.5 Serum biomarkers

Before surgery, there were no statistically significant differences in the serum levels of ET-1, MMP-9, AOPP, Cor, and NE between the two groups (*p* > 0.05). On postoperative day 7, the levels of ET-1, MMP-9, AOPP, Cor, and NE in the ICP monitoring group (49.71 ± 2.87 pg./mL, 112.35 ± 8.22 μg/L, 38.96 ± 1.77 μmol/L, 18.22 ± 1.08 nmol/L, 81.36 ± 8.22 pg./mL) were all significantly lower than those in the control group (62.98 ± 5.22 pg./mL, 145.28 ± 13.78 μg/L, 58.47 ± 3.29 μmol/L, 23.87 ± 1.67 nmol/L, 93.71 ± 8.96 pg./mL, all *p* < 0.05) (see Table 4).

**Table 4:** Comparison of serum biomarkers between the two groups.

Biomarker	Time	ICP monitoring group	Control group	*t*	*P*
Number of cases	–	57	57	–	–
ET-1 (pg/mL)	Before surgery	85.34 ± 10.21	84.97 ± 10.56	0.19	0.849
	After treatment	49.71 ± 2.87	62.98 ± 5.22	−16.52	<0.001
MMP-9 (μg/L)	Before surgery	198.44 ± 21.35	196.88 ± 22.10	0.37	0.710
	On postoperative day 7	112.35 ± 8.22	145.28 ± 13.78	−14.79	<0.001
AOPP (μmol/L)	Before surgery	72.15 ± 5.64	71.89 ± 5.78	0.24	0.811
	On postoperative day 7	38.96 ± 1.77	58.47 ± 3.29	−38.42	<0.001
Cor (nmol/L)	Before surgery	28.56 ± 2.45	28.23 ± 2.61	0.70	0.486
	On postoperative day 7	18.22 ± 1.08	23.87 ± 1.67	−19.66	<0.001
NE (pg/mL)	Before surgery	115.60 ± 12.35	114.87 ± 12.77	0.31	0.758
	On postoperative day 7	81.36 ± 8.22	93.71 ± 8.96	−7.35	<0.001

### 3.6 Activity of daily living

**Figure 2:**
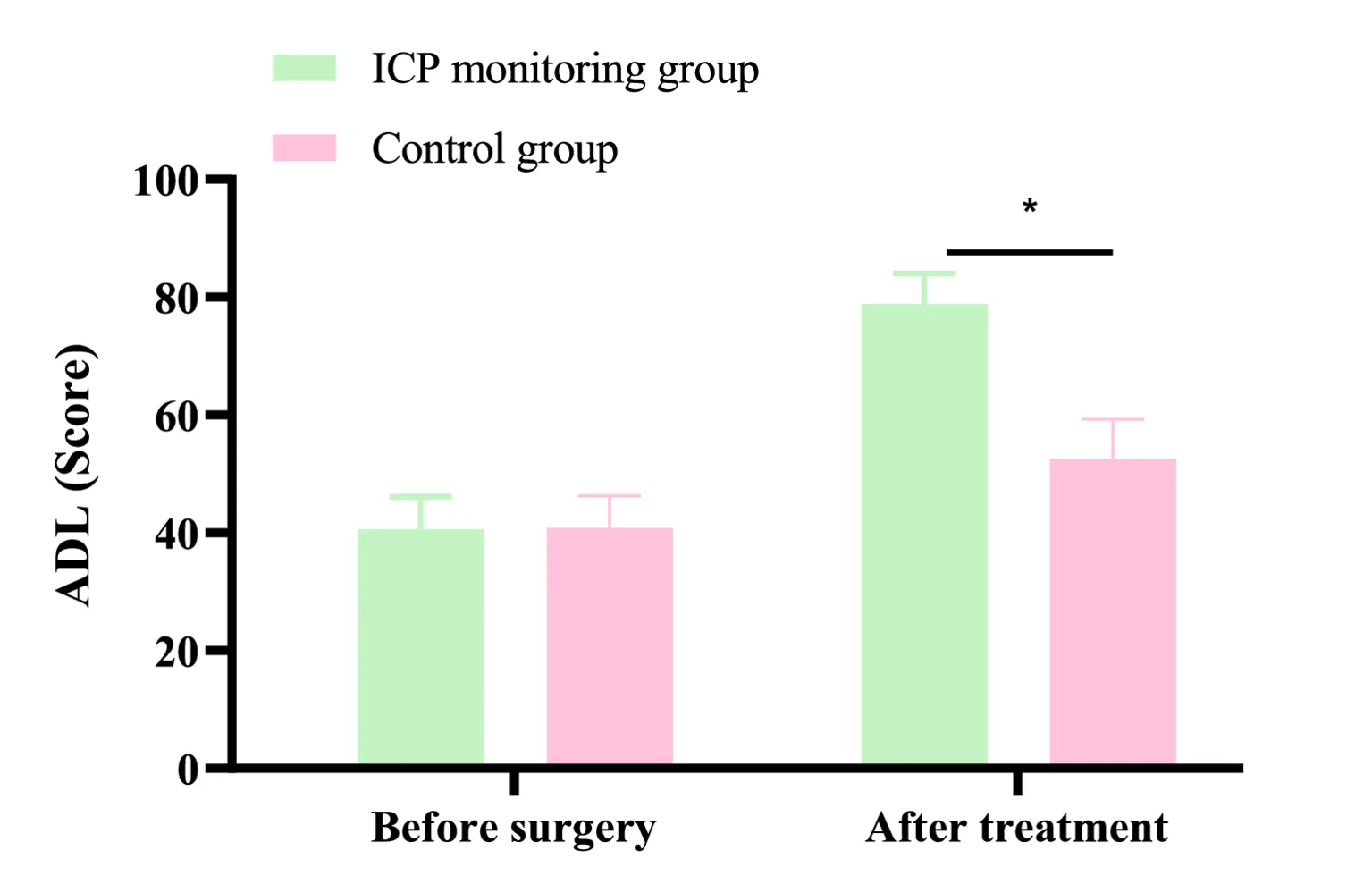
Modified Barthel Index (ADL) scores before treatment and at 3 months in the ICP monitoring and control groups. Bars represent mean ± standard deviation. *p < 0.05 for the between-group comparison at 3 months.

Before surgery, there was no statistically significant difference in ADL scores between the two groups (*p* > 0.05). At 3 months postoperatively, the ADL score in the ICP monitoring group was 78.85 ± 5.17, which was significantly higher than 52.54 ± 6.78 in the control group (*t* = 22.61, *p* < 0.001) (see Figure 2).

### 3.7 Complications

**Figure 3:**
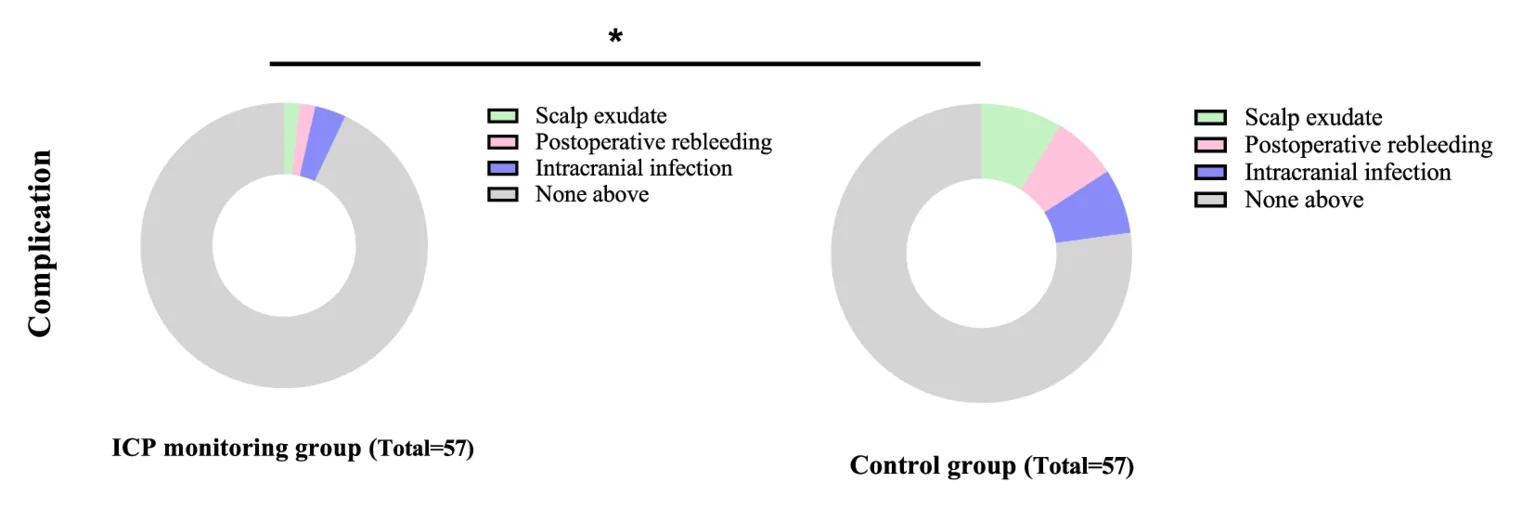
Postoperative complications by type in the ICP monitoring and control groups. Values are counts. *p < 0.05 for the between-group comparison of the overall complication rate.

In the ICP monitoring group, complications were classified according to the external ventricular drain site or the hematoma puncture site. In the ICP monitoring group, there was 1 case of scalp exudation, 1 case of postoperative rebleeding, and 2 cases of intracranial infection, with a total incidence of 7.02% (4/57). In the control group, there were 5 cases of scalp exudation, 4 cases of postoperative rebleeding, and 4 cases of intracranial infection, with a total incidence of 22.81% (13/57). The difference in the overall complication rate between the two groups was statistically significant (*χ*^2^ = 6.09, *p* = 0.014) (see Figure 3).

## 4 Discussion

In this retrospective propensity score-matched cohort, the addition of continuous lateral ventricular ICP monitoring to minimally invasive hematoma evacuation was associated with a higher 3-month favorable outcome rate, greater neurological improvement, better functional independence, and a lower overall complication rate. Although these findings are clinically encouraging, the observational design does not permit causal conclusions, and the EVD intervention combined both ICP monitoring and therapeutic cerebrospinal fluid drainage, which cannot be completely separated.

With regard to surgical indicators, the ICP monitoring group required additional steps including lateral ventricular puncture, subcutaneous tunneling and fixation of the catheter, and calibration of the ICP probe, resulting in an operation time approximately 50 min longer than that of the control group. However, no significant differences were observed in intraoperative blood loss or evacuated hematoma volume, indicating that this procedure did not add traumatic risk or interfere with the core evacuation efficiency. More importantly, the hematoma evacuation time in the ICP monitoring group was shortened by nearly 4 days, and the length of hospital stay was reduced by approximately 5 days. This accelerated recovery may be attributable to the dual function of the EVD: while continuously recording ICP, it also allowed controlled drainage of cerebrospinal fluid, thereby alleviating perihematomal edema and providing favorable mechanical conditions for residual hematoma absorption (9–11). In addition, real-time pressure data enabled the transition of hyperosmolar therapy from empirical administration to a more targeted approach, avoiding delayed ICP control or excessive dehydration (12, 13). Nevertheless, these large differences in process measures should be interpreted cautiously because the decision to remove drains and the timing of discharge may have been influenced by the treating team’s confidence in the monitored patients rather than by biological recovery alone.

In terms of neurological outcome, the 3-month favorable outcome rate was significantly higher in the ICP monitoring group than in the control group, and both the CSS and NIHSS scores demonstrated greater improvement. The use of the Glasgow Outcome Scale rather than a composite “favorable outcome rate” was intended to improve comparability with the neurocritical care literature; however, this remains a relatively coarse ordinal endpoint, and future studies should incorporate the modified Rankin Scale as the preferred functional outcome measure. The observed clinical advantage may be explained by the ability of continuous ICP monitoring to detect early upward trends in ICP and trigger proactive interventions, including posture adjustment, cerebrospinal fluid drainage, sedation optimization, and additional hyperosmolar therapy. This prospective management may help maintain ICP below 20 mmHg and cerebral perfusion pressure above 70 mmHg, thereby reducing recurrent brainstem compression and global cerebral ischemia and preserving neurons in the ischemic penumbra (14–16).

The dynamic changes in serum markers provide a molecular-level correlate, but the interpretation must be cautious. Following intracerebral hemorrhage, hematoma compression and local hypoxia stimulate vascular endothelial cells to excessively secrete endothelin-1, triggering cerebral vasospasm and exacerbating microcirculatory disturbance and edema; precision ICP reduction guided by continuous monitoring may relieve mechanical stretch on vessels, improve perfusion, and thereby suppress its abnormal elevation. The expression of matrix metalloproteinase-9 is closely associated with blood–brain barrier disruption and inflammatory infiltration. When ICP tends to stabilize, the stimulation from mechanical stress and inflammatory factors is attenuated, and the activity of this enzyme decreases, which helps maintain barrier integrity and interrupts the vicious cycle of “edema—inflammation—secondary injury” (17–19). Advanced oxidation protein products are driven by oxidative stress, while cortisol and norepinephrine reflect activation of the sympathetic-adrenal axis. However, because biomarkers were measured only once on postoperative day 7, the observed differences may reflect better overall clinical recovery rather than a direct molecular effect of ICP-guided therapy, and no time-course or causal relationship can be established from these data.

The improvement in postoperative ADL scores is a direct reflection of neurological recovery and overall physical amelioration. The ADL score in the ICP monitoring group was approximately 26 points higher than that in the control group, indicating that patients achieved greater independence in basic daily activities including eating, dressing, toileting, and ambulation. This functional improvement is likely multifactorial, reflecting better motor control, reduced complications, shorter immobilization, and earlier entry into rehabilitation rather than a single mechanistic pathway (20–24).

In terms of safety, the total complication rate in the ICP monitoring group in this study was only 7.02%, significantly lower than the 22.81% in the control group. Intuitively, lateral ventricular puncture and catheterization are invasive procedures that might introduce additional risks of infection and mechanical injury. However, the actual data demonstrate that, under stringent aseptic technique, subcutaneous tunneling, and standardized nursing care, the absolute number of intracranial infections did not increase. In contrast, the control group, lacking real-time ICP guidance, often relied on more aggressive repeated osmotic therapy; fluctuations in intravascular volume and intracranial pressure may have increased the risk of postoperative rebleeding and scalp exudation. With continuous monitoring, the ICP monitoring group was able to adopt a more rational and stepwise approach to hyperosmolar therapy, potentially reducing abrupt changes in blood pressure and ICP. Simultaneously, the ventricular drainage catheter may have accelerated wound healing by draining inflammatory cerebrospinal fluid and reducing local tissue tension, thereby decreasing scalp exudation (25–28). However, complication events were few, and the study had limited power to detect differences in rare safety endpoints.

This study has several important limitations. First, it is a single-center retrospective design; although propensity score matching was used to balance known baseline covariates, residual confounding from unmeasured factors such as postoperative ICU management, timing and indication for ICP monitoring, and specific treatment responses cannot be excluded. Second, the sample size was relatively small, and the number of deaths was insufficient for reliable separate reporting. Third, outcomes were not analyzed separately by hemorrhage location because of the small number of lobar cases, which limits conclusions about treatment effect modification across basal ganglia, thalamic, and lobar hemorrhages. Fourth, the EVD intervention combined continuous ICP monitoring with therapeutic cerebrospinal fluid drainage, and the relative contribution of each component cannot be determined. Fifth, serum biomarkers were measured only at baseline and on postoperative day 7, precluding analysis of their dynamic trajectories. Finally, follow-up was limited to 3 months, and long-term cognitive, functional, and survival outcomes require further investigation. Future multicenter prospective randomized controlled trials should include predefined ICP treatment protocols, systematic mRS and mortality reporting, serial biomarker sampling, and subgroup analyses by hematoma location.

In summary, ICP monitoring combined with hematoma evacuation was associated with accelerated hematoma clearance, shorter hospital stay, better 3-month functional outcome, lower serum levels of ET-1, MMP-9, AOPP, and stress hormones, and fewer complications in this retrospective propensity score-matched cohort. However, these findings should be interpreted as hypothesis-generating rather than confirmatory because of the observational design and the combined monitoring and drainage effects of the EVD.

## Data Availability

The raw data supporting the conclusions of this article will be made available by the authors, without undue reservation.
